# Functionalization of Graphene Oxide with Low Molecular Weight Poly (Lactic Acid)

**DOI:** 10.3390/polym10020177

**Published:** 2018-02-12

**Authors:** Mingwei Yuan, Yike Chen, Minglong Yuan, Hongli Li, Xiansong Xia, Chengdong Xiong

**Affiliations:** 1 Chengdu Institute of Organic Chemistry, Chinese Academy of Sciences, Chengdu 610041, China; yuanmw96@gmail.com (M.Y.); psxxs1982@gmail.com (X.X.); 2 University of Chinese Academy of Sciences, Beijing 100049, China; 3 Engineering Research Center of Biopolymer Functional Materials of Yunnan, Yunnan Minzu University, Kunming 650500, China; chenyike1992@gmail.com (Y.C.); yml@188.com (M.Y.); hongli8302@gmail.com (H.L.)

**Keywords:** GO-*g*-PLLA, composite materials, water vapor, oxygen permeabilities

## Abstract

In this paper, the hydroxyl groups on the surface of graphene oxide (GO) were used to initiate the ring-opening polymerization of a lactic acid *O*-carboxyanhydride. GO grafted with poly (l-lactic acid) molecular chains (GO-*g*-PLLA) was prepared. Lactic acid *O*-carboxyanhydride has a higher polymerization activity under mild polymerization conditions. Thus, the functionalization of the polymer chains and obtaining poly (lactic acid) (PLLA) was easily achieved by ring-opening polymerization with 4-dimethylaminopyridine (DMAP) as the catalyst. The results showed that with this method, PLLA can be rapidly grafted to the surface of GO in one step. As a result, the chemical structure of the GO surface was altered, improving its dispersion in organic solvents and in a PLLA matrix, as well as its bonding strength with the PLLA interface. We then prepared GO/PLLA and PLLA/GO-*g*-PLLA composite materials and investigated the differences in their interfacial properties and mechanical properties. GO-*g*-PLLA exhibited excellent dispersion in the PLLA matrix and formed excellent interfacial bonds with PLLA through mechanical interlocking, demonstrating a significant enhancement effect compared to PLLA. The water vapor and oxygen permeabilities of the GO-*g*-PLLA/PLLA composite decreased by 19% and 29%, respectively.

## 1. Introduction

Poly (lactic acid) (PLLA) is receiving considerable attention for conventional uses, such as a packaging material, for the production of agricultural film, and more recently, as composites for technical applications [1]. However, its wider application has been limited by its relatively slow crystallization rate, poor gas barrier performance, and poor flexibility [1]. To overcome these challenges, various methods (such as Copolymerization and blending) have been used to enhance the comprehensive performance of poly l-lactide (PLLA). The degree of crystallinity and mechanical properties can be changed by blending. Copolymerization can change the performance of many aspects, mainly based on the properties of the graft molecules. For example, a rigid molecule can improve the strength of the copolymer, while the flexible molecule can enhance the tensile properties of the copolymer [1,2]. Graphene and graphene oxide (GO) have been used in PLLA nanocomposites, and graphite or GO can increase the crystallinity and the heat resistant temperature of the nanocomposite [3,4,5,6,7]. In a study of graphene and GO-modified polylactic acid, the main method used was to directly blend graphene with polylactic acid. Feng et al. developed a versatile method by grafting polymers on GO to enhance the properties of nanocomposites [8,9]. Chaobin et al. reported a new type of PLLA-GO nanocomposite and showed that a stereocomplex crystal could be formed between PLLA and GO-*g*-PDLA. The incorporation of GO nanofillers lead to a lower crystallization activation energy of the stereocomplex and a higher crystallinity in solution casting samples [10]. The PLLA-GO nanocomposites were prepared by blending commercial PLLA with GO-*g*-PDLA, in which GO-*g*-PDLA was synthesized via ring-opening polymerization using modified GO as the initiator. According to the above literature, the crystallinity and heat resistance of PLLA can be significantly increased by modifying PLLA with graphene and GO.

Improving the gas barrier performance and flexibility of PLLA for the purpose of packaging and other uses is also necessary. Increasing the compatibility of nano polylactic acid composites and the adhesion force of the interface will improve their gas barrier property and their flexibility. Based on the “like dissolves like” principle, because inorganic nanoparticles are structurally different from polymers, modifying the structure of inorganic nanoparticles to improve their compatibility with polymers is essential. Albertsson et al. [11,12,13,14] used PLLA stereocomplex (SC) particles with a diameter of 300–500 nm to modify PLLA, and this initiated the interfacial crystallization of PLLA, resulting in a clear improvement in compatibility. In particular, the modification of PLLA with GO-PLLA SC composite particles significantly improved the thermal resistance and barrier properties of the material [12,13,14]. Based on the above analysis and the “like dissolves like” principle, this study aimed to prepare high barrier nanomaterial-PLLA membrane materials by modifying PLLA with structurally similar nanomaterials that were functionalized with low molecular weight PLLA. This study provides a new thought process and method to solve the key problem of nanomaterial-PLLA composite materials, and to lay a theoretical foundation for next-generation green food packaging materials.

## 2. Experimental Methods

### 2.1. Materials

#### 2.1.1. Preparation of Raw Materials

Lactic acid *O*-carboxyanhydride (LacOCA) was prepared according to the literature [15,16,17,18]. The L-lactic aqueous solution was provided by Henan Jindan. Lactic Acid, LLC, lithium hydroxide (LiOH), ethanol, *tert*-butyl methyl ether, triphosgene, and tetrahydrofuran were obtained from the Chengdukelong chemical reagent PLLAnt, all with a purity of approximately 99 wt %. GO was provided by Suzhou Carbon Abundance Graphene Science and Technology Co., Ltd., Suzhou, China, with a purity of ~99 wt %, a thickness of 0.6–1.0 nm, a sheet diameter of 0.5–5 µm, with 1–2 layers, and a specific surface area of 1000–1217 m^2^/g. The PLLA was thermal film-grade PLLA from NatureWorks, LLC.

#### 2.1.2. Preparation of GO-*g*-PLLA

**Scheme 1:**
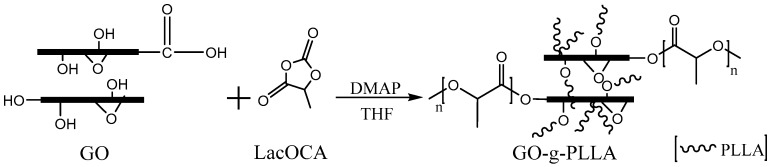
Synthetic route of graphene oxide (GO) grafted with poly l-lactic acid molecular chains (GO-*g*-PLLA).

Tetrahydrofuran (THF) (150 mL) and 0.067 g of GO (lactic acid *O*-carboxyanhydride of 0.5/100) were added to a 250-mL single-neck flask and sonicated for 1 h to evenly disperse the GO in the THF. LacOCA (14 g) was added to the above solution at room temperature and stirred until dissolved. After the solution became clear, 0.046 g of 4-dimethylaminopyridine (DMAP) (1/300 of the amount of LacOCA) was added and stirred at room temperature overnight until no bubbles were present. Once the reaction was complete, the solution was concentrated and dried at 30 °C. Then, chloroform was used to dissolve the polymerization products. At room temperature for 12 h, the insoluble substances were removed by centrifugation. The filtrate of homogeneous phase was precipitated by excessive ethanol to achieve solidity. The solids were filtered and dried under vacuum at 60 °C for 8 h. The product gained was GO-*g*-PLLA (Scheme 1). As a contrast, PLA and GO are also dissolved in chloroform, and at room temperature for 12 h, the insoluble substances were removed by centrifugation. The filtrate of homogeneous phase was precipitated by excessive ethanol to achieve solidity. The solids were filtered and dried under vacuum at 60 °C for 8 h. The product gained was not GO/PLLA blend compound, it was PLA. Filtration after centrifugation was dried using freeze-drying method to obtain GO.

#### 2.1.3. Preparation of GO-*g*-PLLA/PLLA Composite Material

A specific amount of GO-*g*-PLLA (4, 8, or 12 g) was mixed with 800 g of PLLA. Chloroform (2000 mL) was then added and mechanically stirred to dissolve the mixture. After the solution became clear, the solution was sonicated for 2 h. Then, 5000 mL of ethanol was added to the solution to precipitate a gray-white solid that was vacuum-dried at 60 °C for 8 h to obtain GO-*g*-PLLA/PLLA composites containing 0.5%, 1%, and 1.5% GO-*g*-PLLA.

#### 2.1.4. Instruments and Characterization

Nuclear magnetic resonance (NMR), infrared (IR), TGA, and X-ray photoelectron spectroscopy (XPS) were conducted to determine the changes in the chemical characteristics of GO and GO-*g*-PLLA. NMR spectra were recorded with Bruker 400 MHz spectrometers at 25 °C. Chemical shifts for ^1^H NMR spectra were referenced internally using the residual solvent resonances and tetramethylsilane (TMS) as an internal reference. The solvent was used in the deuterium chloroform, and with a solubility of 10 mg/mL. the dissolution was not precipitation. it was limpid. The molecular weight of free PLLA was determined using gel permeation chromatography (GPC) at 40 °C using THF as the eluent with a flow rate of 1 mL/min. The system equipped with a Waters 515 pump and a Waters 2414 refractive index detector. IR (Bruker Tensor 37) spectra were recorded from 600 to 4000 cm^−1^ with a resolution of 2 cm^−1^ and 32 scans. TGA curves were obtained from a Mettler SDTA851e. The samples were heated from 25 to 800 °C at a rate of 10 °C/min in an aluminum crucible under 50 mL/min of nitrogen purging. XPS measurements were performed using a PHI5000 Versaprobe-II System Auger electron spectrometer (Physical Electronics Company, U.S.) equipped with a hemispherical electron analyzer and a scanning monochromatic AlKa (hm = 1486.6 eV) X-ray source. The particle size distributions of GO and GO-*g*-PLLA in the chloroform were tested using a S3500SI laser particle size analyzer system (Micortrac Co. Ltd., Norristown, PA, USA) with an equivalent sphere model and a measuring range of 0.01 to 2800 μm. To further characterize the dispersions of GO and GO-*g*-PLLA before and after grafting in solvents, we performed dynamic light scattering to measure the particle size and distribution. The solutions had a concentration of 1 mg/mL. Each solution was sonicated for 2 h after dissolving the particles and immediately tested.

The crystallization behaviors of PLLA and nanocomposites were measured by a differential scanning calorimeter (DSC) (214, Netzsch, Selb, Germany) in a nitrogen flow (50 mL/min) with a heating rate of 10 °C/min over a temperature range of 20 to 200 °C. Flexural tests of the PLLA composites were determined by a CMT4104 universal tester (Meites Industrial Systems Co., Ltd. (Wuhu, China)). The length, width, and thickness of the sample were 50, 15 and 1.10 mm, respectively, according to GB/T 1040.3-2006 standard, at a crosshead speed of 50 mm/min. Five different measurements were recorded for each sample. The Water Vapor Permeability (WVP) of the films was gravimetrically determined according to the ASTM E96-95 standard. The films had a thickness of 0.04 ± 0.005 mm and were obtained by casting. The screw insertion temperatures were 160, 175, 175, and 170 °C. The PO_2_ was tested on an Oxy Sense5250I oxygen analyzer.

## 3. Results and Discussion

### 3.1. Characterization of GO-g-PLLA

**Figure 1:**
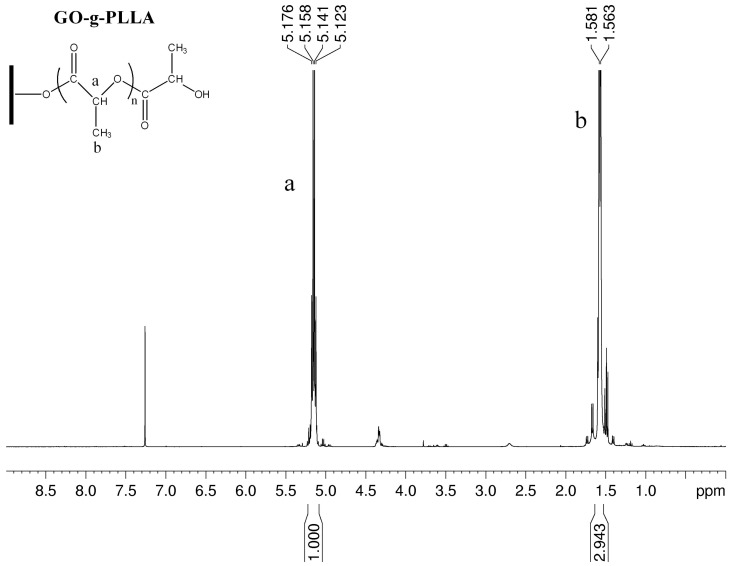
The 400 MHz ^1^H nuclear magnetic resonance (NMR) spectrum of the GO-*g*-PLLA.

Chaobin and Inoue reported the synthesis of GO-*g*-PLLA through the ring-opening polymerization of lactide monomers, initiated by the grafted OH groups on GO and under the catalysis of Sn(Oct)^2^. The synthesis temperature was 120 °C and a metal catalyst was used [10,15]. Considering the biomedical and food potential of PLLAs, studies have focused on metal-free synthetic methods under mild conditions. Bourissou et al. reported the metal-free synthesis of PLLA by ring-opening polymerization of LacOCA. LacOCA exhibited remarkable reactivity compared to lactide. PLLA of controlled molecular weights and narrow polydispersities are typically obtained under mild conditions using DMAP and various protic initiators [16,17,18,19]. The above studies demonstrated the ring-open polymerization of lactide or LacOCA. When there are hydroxyl compounds in the polymerization system, after polymerization, these hydroxyl compounds are linked to PLLA chains through chemical bonds to achieve the modification of PLLA [10,17,18,19]. In this study, we use metal-free synthetic methods to synthesize GO-*g*-PLLA by ring-opening polymerization of LacOCA, as shown in Figure 1.

In our experiment with the preparation of GO-*g*-PLLA, the polymerization was completed. Then chloroform was used to dissolve the polymerization products. After 12 h of placement, we found that the chloroform solution was homogeneous. No insoluble substance was found by centrifugation. The filtrate of homogeneous phase was precipitated by excessive ethanol to achieve solidity. The solids were filtered and dried under vacuum to obtain GO-*g*-PLLA. In the comparison experiment of preparing GO/PLLA blend compound, we found that the chloroform dissolved in GO/PLLA after 12 h statics was divided into two phases. Because GO is insoluble in chloroform, it will be precipitated from the solution. After centrifuge separation, the insoluble substance obtained is pure GO. The compounds dissolved in chloroform were found to be pure PLA after the same process. The two experiments also showed that free GO could be separated from GO-*g*-PLLA or PLLA by chloroform dissolution and centrifugation. If GO/PLLA blend compound is to be prepared, the GO and PLA cannot be centrifuged after mixing in chloroform; GO/PLLA can be obtained by direct freeze-drying.

To confirm that the GO-*g*-PLLA has a PLA structure unit, we performed magnetic characterization on the GO-*g*-PLLA polymers. From Figure 1, the characteristic peaks of PLLA appeared at 1.5 and 5.1 ppm, which agree with data reported in the literature [20,21,22,23,24,25] and belong to the characteristic proton peaks for the methine (a) and methyl (b) groups on the PLLA chain. From the NMR spectrum of the GO-*g*-PLLA, 4.35 ppm corresponds to the methine group –CH– located at the end of the PLLA chain, which is adjacent to the terminal hydroxyl groups of PLLA. This finding is consistent with that of Sun and He [10], in which GO-*g*-PLLA was prepared by the ring-opening polymerization of lactide.

**Figure 2:**
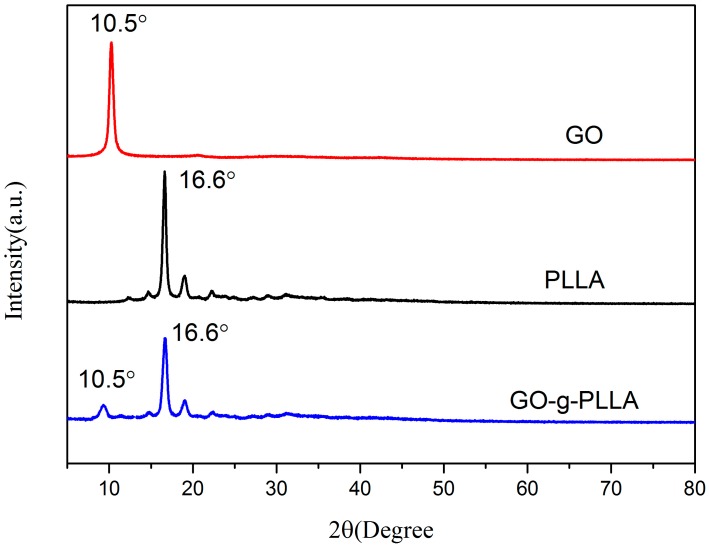
The XRD patterns of pure GO, PLLA, and GO-*g*-PLLA.

To confirm that the GO-*g*-PLLA has a GO structure unit, we performed XRD on the GO-*g*-PLLA polymers. Figure 2 shows the XRD patterns of pure GO, PLLA, and GO-*g*-PLLA composite. As seen, the most intense diffraction peak of PLLA at 2θ = 16.6°, the diffraction peak of GO at 2θ = 10.5°. For the GO-*g*-PLLA composite, the location and shape of the diffraction peaks of the three samples were similar to those of pure PLLA and GO. As the sample was dissolved by chloroform and separated by centrifuge, the results thus show that the PLLA molecular chains were successfully grafted to the surface of GO.

**Figure 3:**
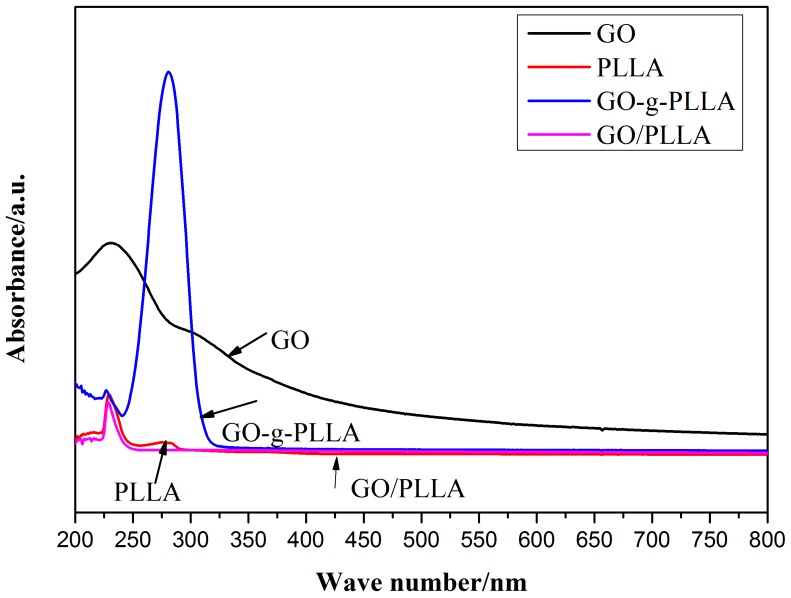
UV/Vis spectra of GO, PLLA, GO-*g*-PLLA, and GO/PLLA.

In order to further confirm that the GO-*g*-PLLA has a GO structure unit, we performed UV/Vis characterization on the GO-*g*-PLLA polymers. Figure 3 shows the UV/Vis spectra of PLLA, GO, GO-*g*-PLLA, and GO/PLLA. The UV/Vis spectrum of PLLA, GO-*g*-PLLA, and GO/PLLA was observed in the DCM solution, while those of PLLA, GO-*g*-PLLA, and GO/PLLA were in the solution. GO-*g*-PLLA shows very broad absorption with continuously decreasing intensity ranged from 220 to 330 nm. On the other hand, PLLA and GO-*g*-PLLA shows the absorption in the range from 250 to 330 nm, and no absorption peak is observed in the 330 to 800 nm range. Further, PLLA, GO-*g*-PLLA, and GO/PLLA shows characteristic peaks in the wave length region shorter than 250 nm, while no evident absorption in the higher wave length region. In the absorption spectrum in the range from 220 to 320 nm, the GO-*g*-PLLA shows absorption with special features characteristics for both PLLA and GO, indirectly indicating that the PLLA chain was grafted onto the surface of GO [15].

### 3.2. Determination of Molecular Weight by Gel Permeation Chromatography (GPC)

We estimated the grafting by testing the molecular weight of the free PLLA generated in the reaction of the molecular length of PLLA to the graphene surface [26,27]. The GO-*g*-PLLA polymer had sharp, unimodal distributions, indicating that GO had completely copolymerized with lactide and that no lactide homopolymerization had occurred. The average molecular weight was 15,000 g/mol, and the polydispersity coefficient was 1.09.

### 3.3. Infrared (IR) Spectroscopy

**Figure 4:**
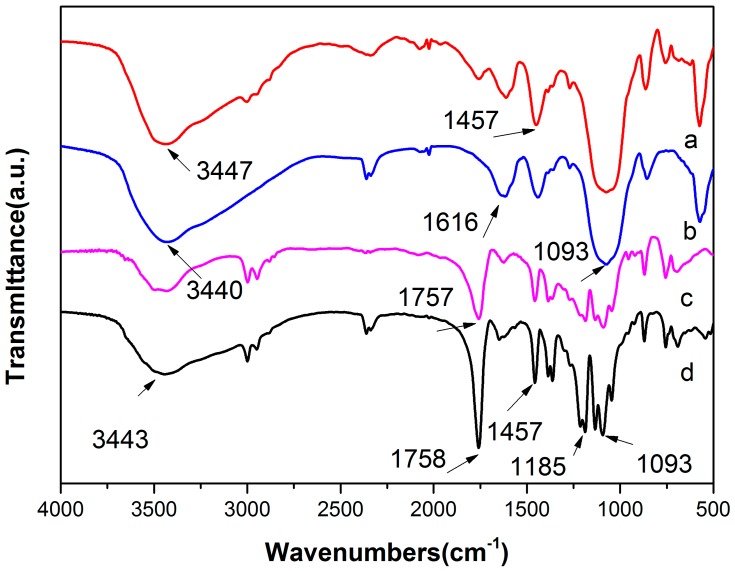
Fourier transform IR (FTIR) spectra of (**a**) PLLA, (**b**) GO, (**c**) GO/PLLA ,and (**d**) GO-*g*-PLLA.

Figure 4 shows the Fourier transform IR (FTIR) spectra of the polymers, in which the typical polyester absorption peaks appeared. In addition, the PLLA, GO, and GO-*g*-PLLA IR measurements detected changes in the chemical functional groups upon grafting, as shown in Figure 4. The peaks at 3447, 3440, and 3443 cm^−1^ belong to the O–H stretching peaks in PLLA, GO, and GO-*g*-PLLA, respectively. The peak at 1758 cm^−1^ corresponds to the stretching vibration peak of C=O in the ester bond in the PLLA chain (Figure 4a,d) [28,29]. A significantly stronger C=O stretching vibration peak appeared at 1758 cm^−1^ for GO-*g*-PLLA, which was likely caused by the grafting of the PLLA molecular chains onto the surface of GO. Therefore, the hydroxyl groups on the surface of GO initiated the ring-opening of the lactic acid *O*-carboxyanhydride and the esterification with carboxyl groups [30]. The GO/PLLA blends were also tested by infrared contrast, and the GO/PLLA curves of the blends were found to be different from those of the copolymer (Figure 4d). Figure 4d is consistent with the findings of Sun and He [10], in which GO-*g*-PLLA was prepared by the ring-opening polymerization of lactide. Furthermore, the characteristic peaks of PLLA, including the stretching vibration of C–CH_3_, the bending vibration of –CH_3_, and the asymmetric bending vibration of –CH_3_, appeared at 1093, 1185, and 1457 cm^−l^, respectively [15,28,31,32,33], in the spectrum of GO-*g*-PLLA. The results thus show that the PLLA molecular chains were successfully grafted onto the surface of GO.

### 3.4. Thermogravimetric Analysis (TGA)

**Figure 5:**
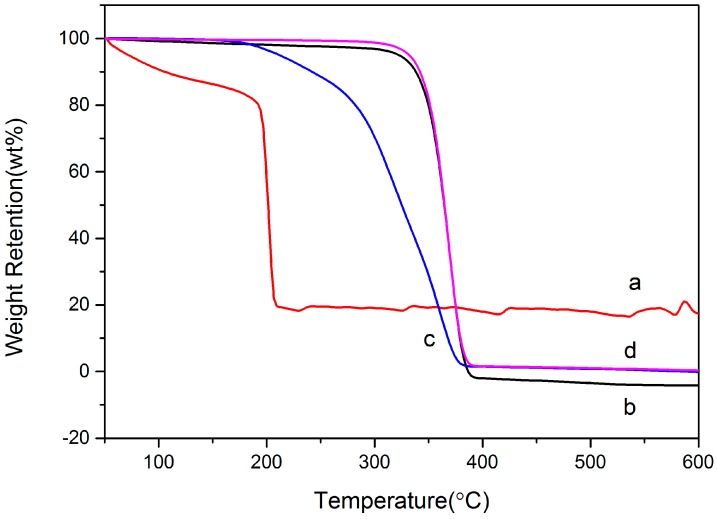
TGA curves of (**a**) GO, (**b**) PLLA, (**c**) GO-*g*-PLLA, and (**d**) GO/PLLA.

From Figure 5, the weight loss of GO appeared in the temperature range of 50 to 200 °C. The weight loss at 50–100 °C was due to the evaporation of physical water in GO, and the weight loss at 100–200 °C was caused by the decomposition of hydroxyl and carboxyl groups on the surface of GO to carbon monoxide (CO), carbon dioxide (CO_2_), and water vapor [34,35,36]. The weight loss of GO-*g*-PLLA appeared at 220–380 °C, which can be divided into two regions. The first region that occurred at 220–280 °C was attributed to the degradation of the residual oxygen-containing functional groups on GO; the other region at 280–380 °C was caused by the degradation of PLLA grafted to the surface of GO [26,34,35]. The PLLA weight loss mainly appeared at 340–380 °C. Based on the literature and according to the TGA curves, the grafting ratio of PLLA to the surface of GO was approximately 60.2 wt % [37] for the in-situ ring-opening polymerization of the lactic acid *O*-carboxyanhydride. The weight loss of GO-*g*-PLLA was between those of GO and PLLA. We also performed a TG analysis on GO/PLLA blends, and the results showed that a significant difference existed compared to the copolymer of GO-*g*-PLLA. The results thus show that the PLLA molecular chains were successfully grafted to the surface of GO.

### 3.5. X-ray Photoelectron Spectroscopy (XPS)

**Figure 6:**
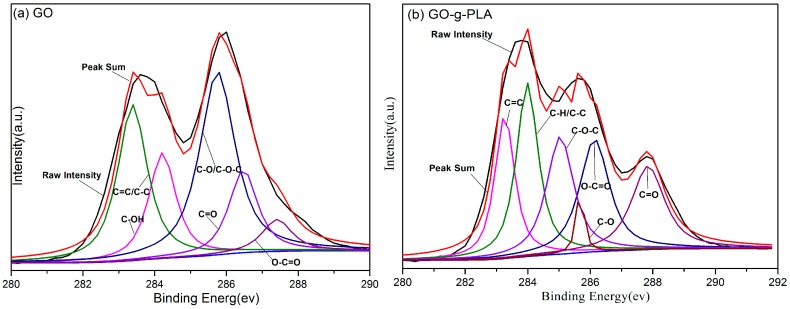
X-ray photoelectron spectroscopy (XPS) curves of (**a**) GO and (**b**) GO-*g*-PLLA.

To further characterize the changes in the chemical functional groups on GO, we performed XPS before and after grafting. The C 1s peaks before and after grafting are shown in Figure 6. The areas of the peaks for GO before grafting were as follows: the C=C/C–C peak (283.38 eV) was 26.1143%, the C–OH peak (284.21 eV) was 16.7404%, the C–O peak (285.75 eV) was 37.8132%, the C=O peak (286.47 eV) was 13.7880%, and the O–C=O peak (287.41 eV) was 5.5441% [38,39]. The C 1s peaks after grafting are shown in Figure 4b. The peak area of –OH for GO changed to 5.5441%, exhibiting a substantial decrease. The peak areas of C–H and O–C=O substantially increased to 17.2537% and 16.8664%, respectively [36]. The results show that, as the initiator, the hydroxyl groups (–OH) on the surface of GO initiated the ring-opening polymerization of LacOCA with the assistance of a catalyst, thus grafting the polymer to the surface of GO [40]. As the reaction progressed, the surface of GO was almost completely covered by a layer of PLLA molecular chains. The test results show that the carbon to oxygen ratio decreased from 2.16 for GO to 1.67 for GO-*g*-PLLA. This is because PLLA contains more oxygen that GO, which agrees with the reports in the literature [37].

### 3.6. Characterization of Physical Properties

**Figure 7:**
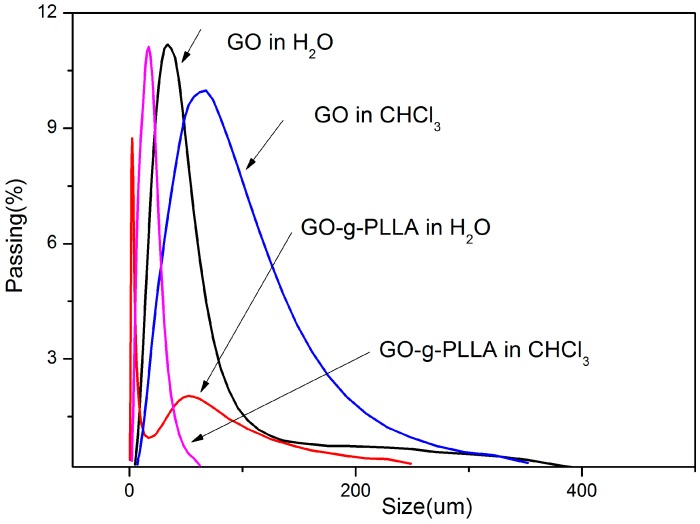
Laser size and shape analysis of GO and GO-*g*-PLLA.

To further characterize the dispersions of GO and GO-*g*-PLLA before and after grafting in solvents, we used dynamic light scattering to measure the particle size and distribution. The solutions had a concentration of 1 mg/mL. Each solution was sonicated for 2 h after dissolving the particles and immediately tested. The results (Figure 7) show that the particle diameters of GO and were 108.5 µm in H_2_O and 163.8 µm in chloroform. Thus, the dispersion of GO in chloroform was significantly better than in water. The results indicate that GO existed as a stable single layer or as few layers in water [41]. Because of the strong van der Waals’ force between the GO nanosheets, GO tends to agglomerate. Therefore, the particle size and size distribution of GO in solvents can reflect its dispersion in solvents [42]. The particle size of GO-*g*-PLLA dissolved in water was 81.8 µm, exhibiting a very wide particle size dispersion. The results show that after the ring-opening polymerization of lactic acid *O*-carboxyanhydride, the hydrophobic PLLA was successfully grafted onto the surface of GO, thus making GO-*g*-PLLA a hydrophobic material, exPLLAining its poor dispersion in water. A particle size of 30.59 µm in chloroform indicated that the dispersion of GO-*g*-PLLA in chloroform was substantially better than that in water. After grafting, GO was transformed from a hydrophilic into a hydrophobic material. Therefore, improving the dispersion of GO in chloroform is possible by obtaining functional GO-*g*-PLLA through the ring-opening polymerization of lactic acid *O*-carboxyanhydride initiated by the hydroxyl groups on the GO surface.

**Figure 8:**
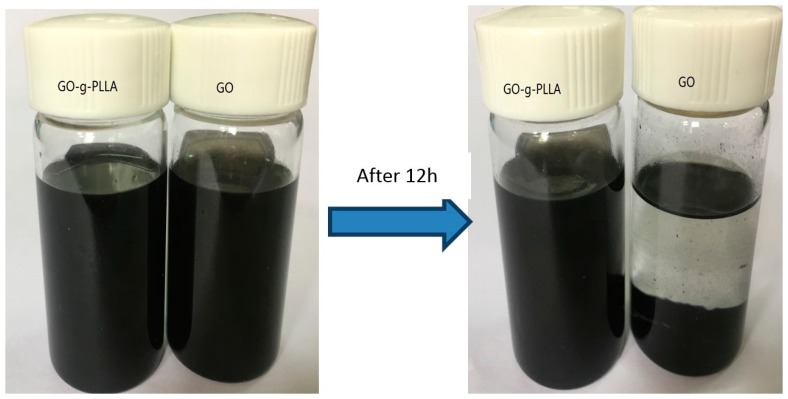
Dispersions of GO and GO-*g*-PLLA in chloroform.

To further confirm the solubility of GO and GO-*g*-PLLA in chloroform, we prepared 0.5 mg/mL GO and GO-*g*-PLLA solutions in chloroform. After the solutions were sonicated for 2 h and left standing for 12 h (Figure 8), the solubility was investigated. All the GO precipitated to the bottom of the chloroform after the solution was sonicated and left for 12 h, whereas GO-*g*-PLLA was still evenly dispersed in the chloroform. Similarly, when chloroform was used to dissolve GO and PLLA blends, 12 h later, GO was precipitated, because the dispersion of GO in chloroform is poor. These different behaviors indicate that the PLLA molecular chains grafted to the GO surface very strongly interacted with the solvent, thus increasing the dispersion of GO-*g*-PLLA in chloroform [37].

### 3.7. Differential Scanning Calorimetry (DSC) of PLLA and Its Composites

**Figure 9:**
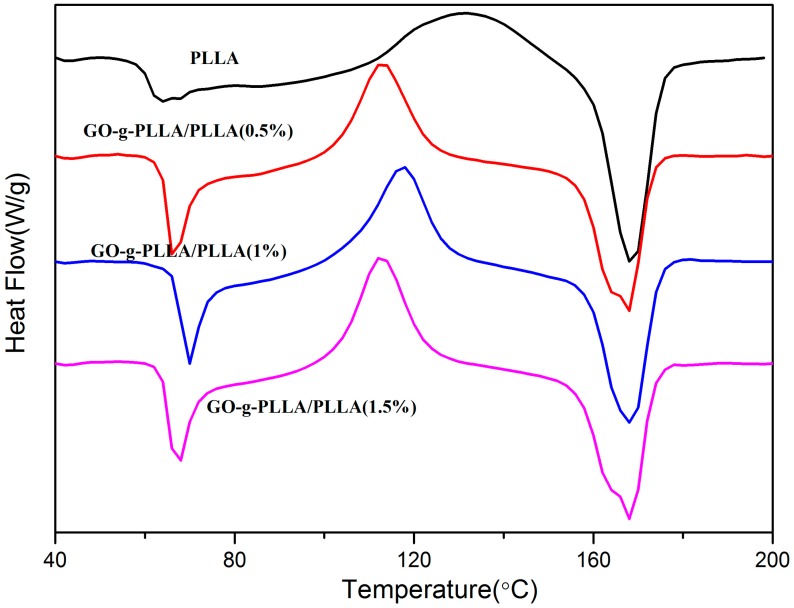
Differential scanning calorimetry (DSC) curves of PLLA and GO-*g*-PLLA/PLLA.

From the DSC curves in Figure 9 and Table 1, the glass transition (Tg) and melting (Tm) temperatures of pure PLLA are 63 and 167.8 °C, respectively. Tg and Tm increased to 68 and 168 °C, respectively, after adding 0.5% GO-*g*-PLLA. Tm increased to 170 °C after adding 1% and 1.5% GO-*g*-PLLA. However, Tg did not increase markedly with further increases in GO-*g*-PLLA content. When 1.5% GO-*g*-PLLA was added, Tg decreased to 62 °C. The increases in Tg and Tm for the nanocomposite were likely due to the increase in the interaction between PLLA and GO and its derivatives. The mechanical interlocking, hydrogen bonding, and/or electrostatic forces restrict the movement between the polymer chains [43,44]. Therefore, the composite with GO-*g*-PLLA exhibited better thermal stability and a higher interfacial adhesion strength than pure PLLA.

In addition, DSC data showed that the addition of the GO-*g*-PLLA composite improved the crystallization rate of PLLA, as shown in Table 1, in which **Δ**Hm (J/g) and Xc represent the enthalpy of melting and degree of crystallinity, respectively. Thus, with the addition of 0.5% GO-*g*-PLLA, Xc increased from 25.6% to 38.3% and continued to increase with increasing amounts of GO-*g*-PLLA composite.

### 3.8. Characterization of the Mechanical Properties of PLLA and Its Composite

**Figure 10:**
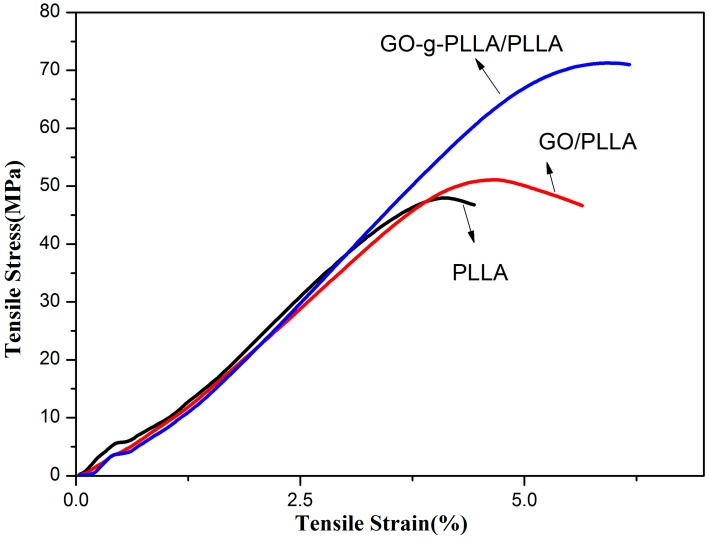
Stress-strain curves of PLLA, GO/PLLA, and GO-*g*-PLLA/PLLA.

Figure 10 shows the tensile strength and breaking strain of PLLA, GO/PLLA, and GO-*g*-PLLA/PLLA. The tensile test results show that the tensile strength and breaking strain of PLLA were 47.97 MPa and 4.44%, respectively, which increased to 51.09 MPa and 5.64% after adding GO and further increased to 71.26 MPa and 6.17% after adding the GO-*g*-PLLA composite. Both copolymers and blends enhance the mechanical properties of PLLA. The GO-*g*-PLLA composite exhibited increased tensile strength and breaking strain of 48.6% and 39%, respectively. The results confirm those of previous reports because of the high specific surface area and high elastic modulus. After dispersion in polymers, graphene and its derivatives can bear loads and thus considerably enhance the mechanical properties of a polymer [45,46,47].

### 3.9. Characterization of the Morphology of PLLA and Its Composite

**Figure 11:**
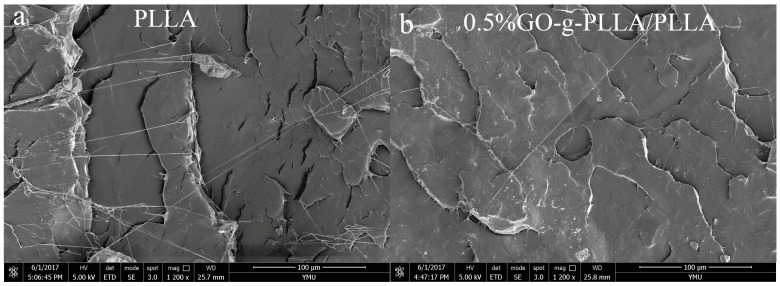
Scanning electron microscopy (SEM) of the tensile fracture surfaces of PLLA and GO-*g*-PLLA/PLLA.

To investigate the dispersion of GO in the composite and the morphology of the fracture surface, we used scanning electron microscopy (SEM) to characterize the fracture surface of PLLA and GO-*g*-PLLA/PLLA, as shown in Figure 11. A large quantity of evident undulations appeared at the fracture surface of PLLA (Figure 11a). In contrast, for GO-*g*-PLLA/PLLA (Figure 11b), the fracture surface was coarser and denser than that of PLLA, indicating that the energy required for fracturing is higher than that for pure PLLA. In addition, the fracture of the composite was different from the brittle fracture of pure PLLA. Conversely, there was no clear agglomeration of graphene on the fracture surface, further indicating that GO-*g*-PLLA was evenly dispersed in PLLA.

### 3.10. Barrier Tests

#### 3.10.1. Water Vapor Permeability (WVP) Test

**Figure 12:**
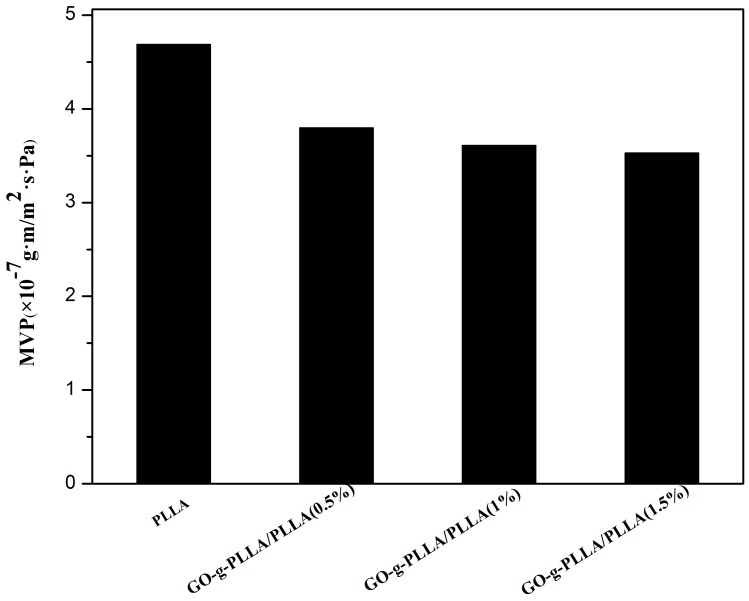
PLLA and GO-*g*-PLLA/PLLA Water Vapor Permeability (WVP) results.

WVP is the most important barrier property for materials. The WVP values of pure PLLA and its nanocomposite films are shown in Figure 12. The films had a thickness of 0.04 ± 0.005 mm and were obtained by casting. The screw insertion temperatures were 160, 175, 175, and 170 °C. The WVP of the pure PLLA thin film was 4.688 × 10^−7^ g·m/m^2^·s·pa. The WVP decreased to 3.797 × 10^−7^ g·m/m^2^·s·pa after adding 0.5% GO-*g*-PLLA, and this value is 19% lower than that of pure PLLA. Further decreases in WVP were not pronounced after adding 1% or 1.5% GO-*g*-PLLA, because with additional GO-*g*-PLLA, the large amount of hydrophilic oxygen-containing functional groups on the GO surface can easily adsorb water, thus increasing the composite absorption capacity of water vapor.

#### 3.10.2. Oxygen Permeability (PO_2_) Test

**Figure 13:**
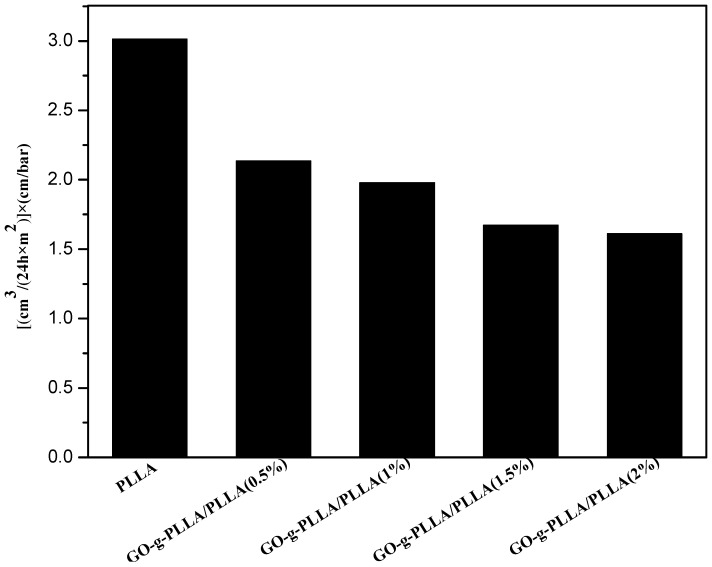
PLLA and GO-*g*-PLLA/PLLA oxygen permeability (PO_2_) results.

The permeability was calculated using Equation (1). The test results are shown in Figure 13. The PO_2_ of PLLA thin film was 3.013 (cm^3^/(24 h × m^2^) × (cm/bar). The oxygen barrier capacity decreased to 2.135 (cm^3^/(24 h × m^2^) × (cm/bar) when 0.5 wt % GO-*g*-PLLA was added, which is a 29.14% reduction. When 2 wt % GO-*g*-PLLA was added, the PO_2_ of the composite thin film decreased to 1.611 (cm^3^/(24 h × m^2^) × (cm/bar), a reduction of 46.53%. In addition, when the amount of GO-*g*-PLLA exceeded 2%, the decrease in PO_2_ was not pronounced.

Permeability = OTR × (thickness/ΔP) = [cm^3^/(m^2^ × 24 h)] × (cm/bar) (1)

## 4. Conclusions

We used hydroxyl groups on the surface of GO to initiate the ring-opening polymerization of lactic acid *O*-carboxyanhydride and successfully grafted PLLA onto the surface of GO surface to obtain the GO-*g*-PLLA composite. Compared to studies in the literature, this method is metal-free and under mild conditions (25 °C). For PLLA with graphene and GO composite materials, the literature focuses on the study of the crystallinity and heat resistance of PLLA. Our study shows that the functional materials prepared by this method largely improve the compatibility and the tensile and barrier properties of the resulting composite materials. When 0.5 wt % of GO-*g*-PLLA was added, the tensile strength and tensile fracture strength of the GO-*g*-PLLA/PLLA composite increased by 48.6% and 39%, respectively, compared to those of PLLA, whereas the water vapor and oxygen permeabilities of the GO-*g*-PLLA/PLLA composite decreased by 19% and 29%, respectively, compared to the neat polymer. Improving the gas barrier performance and flexibility of PLLA for the purpose of packaging and other uses will help to improve the range of its uses in practical applications.

## Figures and Tables

**Table 1:** The glass transition temperature (Tg), melting temperature (Tm), enthalpy of melting (ΔHm), and degree of crystallinity (Xc) of PLLA and GO-*g*-PLLA/PLLA.

Sample	Tg (°C)	Tm (°C)	ΔHm (J/g)	Xc (%)
PLLA	63	167.8	23.84	25.6
GO-*g*-PLLA/PLLA (0.5%)	68	168	35.67	38.3
GO-*g*-PLLA/PLLA (1%)	68	170	39.51	42.4
GO-*g*-PLLA/PLLA (1.5%)	62	170	37.13	39.8

## References

[1] Rasal R.M., Janorkar A.V., Hirt D.E. (2010). Poly(lactic acid) modifications. Prog. Polym. Sci..

[2] Anderson K.S., Schreck K.M., Hillmyer M.A. (2008). Toughening polylactide. Polym. Rev..

[3] Xu J.-Z., Chen T., Yang C.-L., Li Z.-M., Mao Y.-M., Zeng B.-Q., Hsiao B.S. (2010). Isothermal crystallization of poly(l-lactide) induced by graphene nanosheets and carbon nanotubes: A comparative study. Macromolecules.

[4] Murariu M., Dechief A.L., Bonnaud L., Paint Y., Gallos A., Fontaine G., Bourbigot S., Dubois P. (2010). The production and properties of polylactide composites filled with expanded graphite. Polym. Degrad. Stab..

[5] Cao Y., Feng J., Wu P. (2010). Preparation of organically dispersible graphene nanosheet powders through a lyophilization method and their poly(lactic acid) composites. Carbon.

[6] Wang H., Qiu Z. (2012). Crystallization kinetics and morphology of biodegradable poly(l-lactic acid)/graphene oxide nanocomposites: Influences of graphene oxide loading and crystallization temperature. Thermochim. Acta.

[7] Wang H., Qiu Z. (2011). Crystallization behaviors of biodegradable poly(l-lactic acid)/graphene oxide nanocomposites from the amorphous state. Thermochim. Acta.

[8] Liu S., Gordiichuk P., Wu Z.-S., Liu Z., Wei W., Wagner M., Mohamed-Noriega N., Wu D., Mai Y., Herrmann A. (2015). Patterning two-dimensional free-standing surfaces with mesoporous conducting polymers. Nat. Commun..

[9] Liu S., Zhang J., Dong R., Gordiichuk P., Zhang T., Zhuang X., Mai Y., Liu F., Herrmann A., Feng X. (2016). Two-dimensional mesoscale-ordered conducting polymers. Angew. Chem. Int. Ed..

[10] Sun Y., He C. (2012). Synthesis and stereocomplex crystallization of poly(lactide)-graphene oxide nanocomposites. ACS Macro Lett..

[11] Arias V., Odelius K., Hoglund A., Albertsson A.-C. (2015). Homocomposites of polylactide (PLLA) with induced interfacial stereocomplex crystallites. ACS Sustain. Chem. Eng..

[12] Xu H., Wu D., Yang X., Xie L., Hakkarainen M. (2015). Thermostable and impermeable “nano-barrier walls” constructed by poly(lactic acid) stereocomplex crystal decorated graphene oxide nanosheets. Macromolecules.

[13] Xu H., Xie L., Wu D., Hakkarainen M. (2016). Immobilized graphene oxide nanosheets as thin but strong nanointerfaces in biocomposites. ACS Sustain. Chem. Eng..

[14] Xu H., Feng Z.-X., Xie L., Hakkarainen M. (2016). Graphene oxide-driven design of strong and flexible biopolymer barrier films: From smart crystallization control to affordable engineering. ACS Sustain. Chem. Eng..

[15] Hua L., Kai W., Yang J., Inoue Y. (2010). A new poly(l-lactide)-grafted graphite oxide composite: Facile synthesis, electrical properties and crystallization behaviors. Polym. Degrad. Stab..

[16] Kricheldorf H.R., Jonte J.M. (1983). New polymer syntheses. 8. Synthesis and polymerization of l-lactic acid *O*-carboxyanhydride (5-methyl-dioxolan-2,4-dione). Polym. Bull..

[17] Bonduelle C., Martin-Vaca B., Bourissou D. (2009). Lipase-catalyzed ring-opening polymerization of the *O*-carboxylic anhydride derived from lactic acid. Biomacromolecules.

[18] Thillaye du Boullay O., Marchal E., Martin-Vaca B., Cossio F.P., Bourissou D. (2006). An activated equivalent of lactide toward organocatalytic ring-opening polymerization. J. Am. Chem. Soc..

[19] Chen C., Chen L., Chen H., Li H., Yuan M. (2013). Synthesis and solubility of polylactide-*co*-poly(amino acid) random copolymer. Polym. Bull..

[20] Yuan M.L., Li X.H., Xiong C.D., Deng X.M. (1999). Polymerization of lactides and lactones—5. Ring-opening polymerization of epsilon-caprolactone and dl-lactide by rare earth 2-methylphenyl samarium. Eur. Polym. J..

[21] Deng X.M., Yuan M.L., Xiong C.D., Li X.H. (1999). Polymerization of lactides and lactones. II. Ring-opening polymerization of epsilon-caprolactone and dl-lactide by organoacid rare earth compounds. J. Appl. Polym. Sci..

[22] Yuan M.L., Xiong C.D., Li X.H., Deng X.M. (1999). Polymerization of lactides and lactones. III. Ring-opening polymerization of dl-lactide by the (eta(3)-c3h5)(2)sm(mu(2)-cl)(2)(mu(3)-cl)mg(tmed)(mu(2)-cl)mg(tmed) complex. J. Appl. Polym. Sci..

[23] Deng X.M., Yuan M.L., Xiong C.D., Li X.H. (1999). Polymerization of lactides and lactones. IV. Ring-opening polymerization of epsilon-caprolactone by rare earth phenyl compounds. J. Appl. Polym. Sci..

[24] Deng X.M., Yuan M.L., Li X.H., Xiong C.D. (2000). Polymerization of lactides and lactones vii. Ring-opening polymerization of lactide by rare earth phenyl compounds. Eur. Polym. J..

[25] Yuan M.L., Wang Y.H., Li X.H., Xiong C.D., Deng X.M. (2000). Polymerization of lactides and lactones. 10. Synthesis, characterization, and application of amino-terminated poly(ethylene glycol)-*co*-poly(epsilon-caprolactone) block copolymer. Macromolecules.

[26] Fang M., Wang K., Lu H., Yang Y., Nutt S. (2009). Covalent polymer functionalization of graphene nanosheets and mechanical properties of composites. J. Mater. Chem..

[27] Wang J., Shi Z., Ge Y., Wang Y., Fan J., Yin J. (2012). Functionalization of unzipped carbon nanotube via in situ polymerization for mechanical reinforcement of polymer. J. Mater. Chem..

[28] Song W., Zheng Z., Tang W., Wang X. (2007). A facile approach to covalently functionalized carbon nanotubes with biocompatible polymer. Polymer.

[29] Yoon J.T., Lee S.C., Jeong Y.G. (2010). Effects of grafted chain length on mechanical and electrical properties of nanocomposites containing polylactide-grafted carbon nanotubes. Compos. Sci. Technol..

[30] Goffin A.-L., Raquez J.-M., Duquesne E., Siqueira G., Habibi Y., Dufresne A., Dubois P. (2011). From interfacial ring-opening polymerization to melt processing of cellulose nanowhisker-filled polylactide-based nanocomposites. Biomacromolecules.

[31] Yoon J.T., Jeong Y.G., Lee S.C., Min B.G. (2009). Influences of poly(lactic acid)-grafted carbon nanotube on thermal, mechanical, and electrical properties of poly(lactic acid). Polym. Adv. Technol..

[32] Amirian M., Chakoli A.N., Sui J.H., Cai W. (2012). Enhanced mechanical and photoluminescence effect of poly(l-lactide) reinforced with functionalized multiwalled carbon nanotubes. Polym. Bull..

[33] Olalde B., Aizpurua J.M., Garcia A., Bustero I., Obieta I., Jurado M.J. (2008). Single-walled carbon nanotubes and multiwalled carbon nanotubes functionalized with poly(l-lactic acid): A comparative study. J. Phys. Chem. C.

[34] Chen G.X., Kim H.S., Park B.H., Yoon J.S. (2005). Controlled functionalization of multiwalled carbon nanotubes with various molecular-weight poly(l-lactic acid). J. Phys. Chem. B.

[35] Chen L., Xu Z., Li J., Li Y., Shan M., Wang C., Wang Z., Guo Q., Liu L., Chen G. (2012). A facile strategy to prepare functionalized graphene via intercalation, grafting and self-exfoliation of graphite oxide. J. Mater. Chem..

[36] Yang J.-H., Lin S.-H., Lee Y.-D. (2012). Preparation and characterization of poly(l-lactide)-graphene composites using the in situ ring-opening polymerization of PLLA with graphene as the initiator. J. Mater. Chem..

[37] Li W., Xu Z., Chen L., Shan M., Tian X., Yang C., Lv H., Qian X. (2014). A facile method to produce graphene oxide-g-poly(l-lactic acid) as an promising reinforcement for plla nanocomposites. Chem. Eng. J..

[38] Stankovich S., Dikin D.A., Piner R.D., Kohlhaas K.A., Kleinhammes A., Jia Y., Wu Y., Nguyen S.T., Ruoff R.S. (2007). Synthesis of graphene-based nanosheets via chemical reduction of exfoliated graphite oxide. Carbon.

[39] Dreyer D.R., Park S., Bielawski C.W., Ruoff R.S. (2010). The chemistry of graphene oxide. Chem. Soc. Rev..

[40] Sun Y., He C. (2013). Synthesis, stereocomplex crystallization, morphology and mechanical property of poly(lactide)-carbon nanotube nanocomposites. RSC Adv..

[41] Shen J., Hu Y., Shi M., Lu X., Qin C., Li C., Ye M. (2009). Fast and facile preparation of graphene oxide and reduced graphene oxide nanoPLLAtelets. Chem. Mater..

[42] Cao A., Liu Z., Chu S., Wu M., Ye Z., Cai Z., Chang Y., Wang S., Gong Q., Liu Y. (2010). A facile one-step method to produce graphene-cds quantum dot nanocomposites as promising optoelectronic materials. Adv. Mater..

[43] He L., Sun J., Wang X., Fan X., Zhao Q., Cai L., Song R., Ma Z., Huang W. (2012). Unzipped multiwalled carbon nanotubes-incorporated poly(l-lactide) nanocomposites with enhanced interface and hydrolytic degradation. Mater. Chem. Phys..

[44] Leszczynska A., Pielichowski K. (2008). Application of thermal analysis methods for characterization of polymer/montmorillonite nanocomposites. J. Therm. Anal. Calorim..

[45] Suk J.W., Piner R.D., An J., Ruoff R.S. (2010). Mechanical properties of mono layer graphene oxide. ACS Nano.

[46] Ramanathan T., Abdala A.A., Stankovich S., Dikin D.A., Herrera-Alonso M., Piner R.D., Adamson D.H., Schniepp H.C., Chen X., Ruoff R.S. (2008). Functionalized graphene sheets for polymer nanocomposites. Nat. Nanotechnol..

[47] Sengupta R., Bhattacharya M., Bandyopadhyay S., Bhowmick A.K. (2011). A review on the mechanical and electrical properties of graphite and modified graphite reinforced polymer composites. Prog. Polym. Sci..

